# Convolutional Neural Networks-Based MRI Image Analysis for the Alzheimer’s Disease Prediction From Mild Cognitive Impairment

**DOI:** 10.3389/fnins.2018.00777

**Published:** 2018-11-05

**Authors:** Weiming Lin, Tong Tong, Qinquan Gao, Di Guo, Xiaofeng Du, Yonggui Yang, Gang Guo, Min Xiao, Min Du, Xiaobo Qu, The Alzheimer’s Disease Neuroimaging Initiative

**Affiliations:** ^1^College of Physics and Information Engineering, Fuzhou University, Fuzhou, China; ^2^School of Opto-Electronic and Communication Engineering, Xiamen University of Technology, Xiamen, China; ^3^Fujian Key Lab of Medical Instrumentation & Pharmaceutical Technology, Fuzhou, China; ^4^Imperial Vision Technology, Fuzhou, China; ^5^School of Computer & Information Engineering, Xiamen University of Technology, Xiamen, China; ^6^Department of Radiology, Xiamen 2nd Hospital, Xiamen, China; ^7^Fujian Provincial Key Laboratory of Eco-Industrial Green Technology, Nanping, China; ^8^Department of Electronic Science, Xiamen University, Xiamen, China

**Keywords:** Alzheimer’s disease, deep learning, convolutional neural networks, mild cognitive impairment, magnetic resonance imaging

## Abstract

Mild cognitive impairment (MCI) is the prodromal stage of Alzheimer’s disease (AD). Identifying MCI subjects who are at high risk of converting to AD is crucial for effective treatments. In this study, a deep learning approach based on convolutional neural networks (CNN), is designed to accurately predict MCI-to-AD conversion with magnetic resonance imaging (MRI) data. First, MRI images are prepared with age-correction and other processing. Second, local patches, which are assembled into 2.5 dimensions, are extracted from these images. Then, the patches from AD and normal controls (NC) are used to train a CNN to identify deep learning features of MCI subjects. After that, structural brain image features are mined with FreeSurfer to assist CNN. Finally, both types of features are fed into an extreme learning machine classifier to predict the AD conversion. The proposed approach is validated on the standardized MRI datasets from the Alzheimer’s Disease Neuroimaging Initiative (ADNI) project. This approach achieves an accuracy of 79.9% and an area under the receiver operating characteristic curve (AUC) of 86.1% in leave-one-out cross validations. Compared with other state-of-the-art methods, the proposed one outperforms others with higher accuracy and AUC, while keeping a good balance between the sensitivity and specificity. Results demonstrate great potentials of the proposed CNN-based approach for the prediction of MCI-to-AD conversion with solely MRI data. Age correction and assisted structural brain image features can boost the prediction performance of CNN.

## Introduction

Alzheimer’s disease (AD) is the cause of over 60% of dementia cases (Burns and Iliffe, 2009), in which patients usually have a progressive loss of memory, language disorders and disorientation. The disease would ultimate lead to the death of patients. Until now, the cause of AD is still unknown, and no effective drugs or treatments have been reported to stop or reverse AD progression. Early diagnosis of AD is essential for making treatment plans to slow down the progress to AD. Mild cognitive impairment (MCI) is known as the transitional stage between normal cognition and dementia (Markesbery, 2010), about 10–15% individuals with MCI progress to AD per year (Grundman et al., 2004). It was reported that MCI and AD were accompanied by losing gray matter in brain (Karas et al., 2004), thus neuropathology changes could be found several years before AD was diagnosed. Many previous studies used neuroimaging biomarkers to classify AD patients at different disease stages or to predict the MCI-to-AD conversion (Cuingnet et al., 2011; Zhang et al., 2011; Tong et al., 2013, 2017; Guerrero et al., 2014; Suk et al., 2014; Cheng et al., 2015; Eskildsen et al., 2015; Li et al., 2015; Liu et al., 2015; Moradi et al., 2015). In these studies, structural magnetic resonance imaging (MRI) is one of the most extensively utilized imaging modality due to non-invasion, high resolution and moderate cost.

To predict MCI-to-AD conversion, we separate MCI patients into two groups by the criteria that whether they convert to AD within 3 years or not (Moradi et al., 2015; Tong et al., 2017). These two groups are referred to as MCI converters and MCI non-converters. The converters generally have more severe deterioration of neuropathology than that of non-converters. The pathological changes between converters and non-converters are similar to those between AD and NC, but much milder. Therefore, it much more difficult to classify converters/non-converters than AD/NC. This prediction with MRI is challenging because the pathological changes related to AD progression between MCI non-converter and MCI converter are subtle and inter-subject variable. For example, ten MRI-based methods for predicting MCI-to-AD conversion and six of them perform no better than random classifier (Cuingnet et al., 2011). To reduce the interference of inter-subject variability, MRI images are usually spatially registered to a common space (Coupe et al., 2012; Young et al., 2013; Moradi et al., 2015; Tong et al., 2017). However, the registration might change the AD related pathology and loss some useful information. The accuracy of prediction is also influenced by the normal aging brain atrophy, with the removal of age-related effect, the performance of classification was improved (Dukart et al., 2011; Moradi et al., 2015; Tong et al., 2017).

Machine learning algorithms perform well in computer-aided predictions of MCI-to-AD conversion (Dukart et al., 2011; Coupe et al., 2012; Wee et al., 2013; Young et al., 2013; Moradi et al., 2015; Beheshti et al., 2017; Cao et al., 2017; Tong et al., 2017). In recent years, deep learning, as a promising machine learning methodology, has made a big leap in identifying and classifying patterns of images (Li et al., 2015; Zeng et al., 2016, 2018). As the most widely used architecture of deep learning, convolutional neural networks (CNN) has attracted a lot of attention due to its great success in image classification and analysis (Gulshan et al., 2016; Nie et al., 2016; Shin et al., 2016; Rajkomar et al., 2017; Du et al., 2018). The strong ability of CNN motivates us to develop a CNN-based prediction method of AD conversion.

In this work, we propose a CNN-based prediction approach of AD conversion using MRI images. A CNN-based architecture is built to extract high level features of registered and age-corrected hippocampus images for classification. To further improve the prediction, more morphological information is added by including FreeSurfer-based features (FreeSurfer, RRID:SCR_001847) (Fischl and Dale, 2000; Fischl et al., 2004; Desikan et al., 2006; Han et al., 2006). Both CNN and FreeSurfer features are fed into an extreme learning machine as classifier, which finally makes the decision of MCI-to-AD. Our main contributions to boost the prediction performance include: (1) Multiple 2.5D patches are extracted for data augmentation in CNN; (2) both AD and NC are used to train the CNN, digging out important MCI features; (3) CNN-based features and FreeSurfer-based features are combined to provide complementary information to improve prediction. The performance of the proposed approach was validated on the standardized MRI datasets from the Alzheimer’s Disease Neuroimaging Initiative (ADNI – Alzheimer’s Disease Neuroimaging Initiative, RRID:SCR_003007) (Wyman et al., 2013) and compared with other state-of-the-art methods (Moradi et al., 2015; Tong et al., 2017) on the same datasets.

## Materials and Methods

**FIGURE 1:**
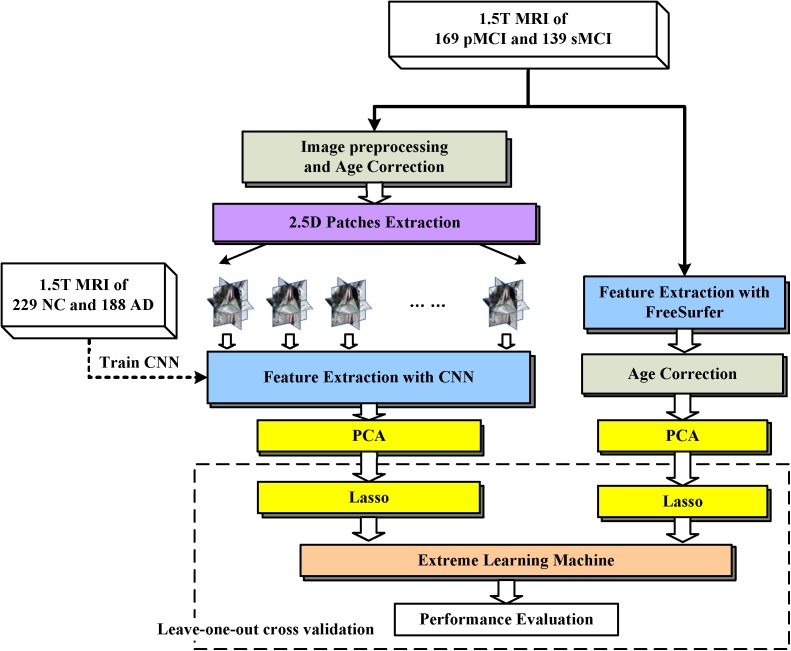
Framework of proposed approach. The dashed arrow indicates the CNN was trained with 2.5D patches of NC and AD subjects. The dashed box indicates Leave-one-out cross validation was performed by repeat LASSO and extreme learning machine 308 times, in each time one different MCI subject was leaved for test, and the other subjects with their labels were used to train LASSO and extreme learning machine.

The proposed framework is illustrated in Figure 1. The MRI data were processed through two paths, which extract the CNN-based and FreeSurfer-based image features, respectively. In the left path, CNN is trained on the AD/NC image patches and then is employed to extract CNN-based features on MCI images. In the right path, FreeSurfer-based features which were calculated with FreeSurfer software. These features, which were further mined with dimension reduction and sparse feature selection via PCA and Lasso, respectively, were concatenated as a features vector and fed to extreme learning machine as classifier. Finally, to evaluate the performance of the proposed approach, the leave-one-out cross validation is then used.

### ADNI Data

Data used in this work were downloaded from the ADNI database. The ADNI is an ongoing, longitudinal study designed to develop clinical, imaging, genetic, and biochemical biomarkers for the early detection and tracking of AD. The ADNI study began in 2004 and its first 6-year study is called ADNI1. Standard analysis sets of MRI data from ADNI1 were used in this work, including 188 AD, 229 NC, and 401 MCI subjects (Wyman et al., 2013). These MCI subjects were grouped as: (1) MCI converters who were diagnosed as MCI at first visit, but converted to AD during the longitudinal visits within 3 years (*n* = 169); (2) MCI non-converters who did not convert to AD within 3 years (*n* = 139). The subjects who were diagnosed as MCI at least twice, but reverse to NC at last, are also considered as MCI non-converters; (3) Unknown MCI subjects who missed some diagnosis which made the last state of these subjects was unknown (*n* = 93). The demographic information of the dataset are presented in Table 1. The age ranges of different groups are similar. The proportions of male and female are close in AD/NC groups while proportions of male are higher than female in MCI groups.

**Table 1:** The demographic information of the dataset used in this work.

	AD	NC	MCIc	MCInc	MCIun
Subjects’ number	188	229	169	139	93
Age range	55–91	60–90	55–88	55–88	55–89
Males/Females	99/89	119/110	102/67	96/43	60/33


*MCIc means MCI converters. MCInc means MCI non-converters, MCIun means MCI unknown.*

### Image Preprocessing

MRI images were preprocessed following steps in Tong et al. (2017). All images were first skull-stripped according to Leung et al. (2011), and then aligned to the MNI151 template using a B-spline free-form deformation registration (Rueckert et al., 1999). In the implementation, we follow the Tong’s way to register images (Tong et al., 2017), showing that the effect of deformable registration with a control point spacing between 10 and 5 mm have the best performance in classifying AD/NC and converters/non-converters. After that, image intensities of the subjects were normalized by deform the histogram of each subject’s image to match the histogram of the MNI151 template (Nyul and Udupa, 1999). Finally, all MRI images were in the same template space and had the same intensity range.

### Age Correction

Normal aging has atrophy effects similar with AD (Giorgio et al., 2010). To reduce the confounding effect of age-related atrophy, age correction is necessary to remove age-related effects, which is estimated by fitting a pixel regression model (Dukart et al., 2011) to the subjects’ ages. We assume there are *N* healthy subjects and *M* voxels in each preprocessed MRI image, and denote **y***_m_*∈**R**^1 × *N*^ as the vector of the intensity values of *N* healthy subjects at *m*th voxel, and α∈**R**^1 × *N*^ as the vector of the ages of *N* healthy subjects. The age-related effect is estimated by fitting linear regression model ***y****_m_* = ω*_m_*α + *b_m_* at *m*th voxel. For *n*th subject, the new intensity of *m*th voxel can be calculated as *y′_mn_* = ω*_m_*(*C*-α*_n_*) + *y_mn_*, where *y_mn_* is original intensity, α*_n_* is age of *n*th subject. In this study, *C* is 75, which is the mean age of all subjects.

### CNN-Based Features

**FIGURE 2:**
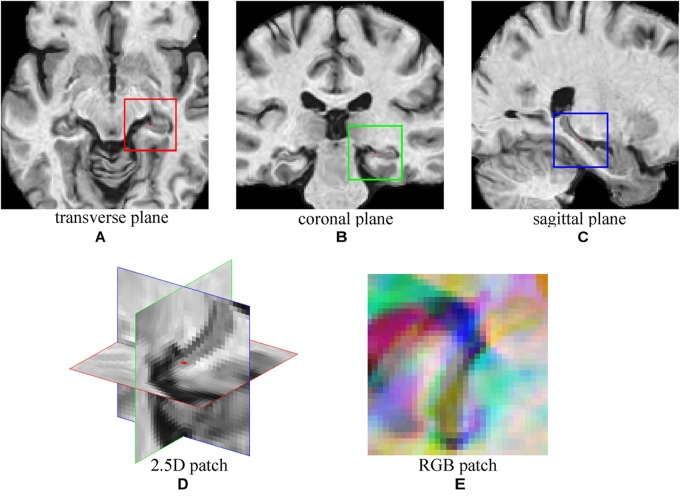
The demonstration of 2.5D patch extraction from hippocampus region. **(A–C)** 2D patches extracted from transverse (red box), coronal (green box), and sagittal (blue box) plane; **(D)** The 2.5D patch with three patches at their spatial locations, red dot is the center of 2.5D patch; **(E)** Three patches are combined into RGB patch as red (red box patch), green (green box patch), and blue (blue box patch) channels.

A CNN was adopted to extract features from MRI Images of NC and AD subjects. Then, the trained CNN was used to extract image features of MCI subjects. To explore the multiple plane images in MRI, a 2.5D patch was formed by extracting three 32 × 32 patches from transverse, coronal, and sagittal plane centered at a same point (Shin et al., 2016). Then, three patches were combined into a 2D RBG patch. Figure 2 shows an example of constructing 2.5D patch. For a given voxel point, three patches of MRI are extracted from three planes and then concatenated into a three channel cube, following the same way of composing a colorful patch with red/green/blue channels that are commonly used in computer vision. This process allows us to mine fruitful information form 3D views of MRI by feeding the 2.5D patch into the typical color image processing CNN network. Data augmentation (Shin et al., 2016) was used to increase training samples, by extracting multiple patches at different locations from MRI images. The choice of locations has three constraints, (1) The patches must be originated in either left or right hippocampus region which have high correlation with AD (van de Pol et al., 2006); (2) There must be at least two voxels distance between each location; (3) All locations were random chosen. With these constraints, 151 patches were extracted from each image and the sampling positions were fixed during experiments. The number of samples was expanded by a factor of 151, which could reduce over-fitting.

**FIGURE 3:**
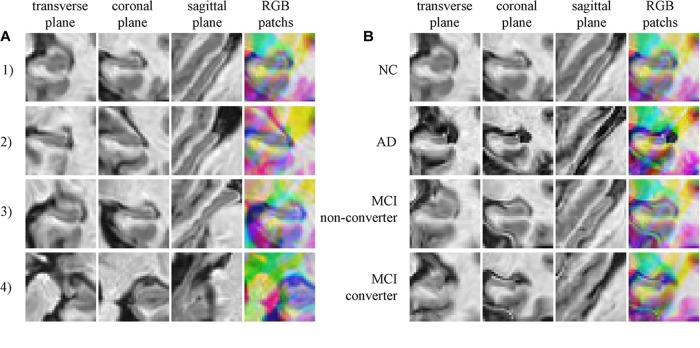
**(A)** Four random chosen 2.5D patches of one subject (who is normal control, female and 76.3 years old), indicating that these patches contain different information of hippocampus; **(B)** The comparison of correspond 2.5D patches of four subjects from four groups, the different level of hippocampus atrophy can be found.

Typically extracted patches are presented in Figure 3. Figure 3A shows four 2.5D patches obtained from one subject. These patches are extracted from different positions and show different portions of hippocampus, which means these patches contain different information of morphology of hippocampus. When trained with these patches that spread in whole hippocampus, CNN learns the morphology of whole hippocampus. Figure 3B shows patches extracted in same position from four subjects of different groups, demonstrating that the AD subject has the most severe atrophy of hippocampus and expansion of ventricle. This implies that obvious differences are existed between AD and NC. However, the MCI subjects have the medium atrophy of hippocampus, and non-converter is more like NC rather than AD, and converter is more similar to AD. The difference between converter and non-converter is smaller than the difference between AD and NC.

**FIGURE 4:**
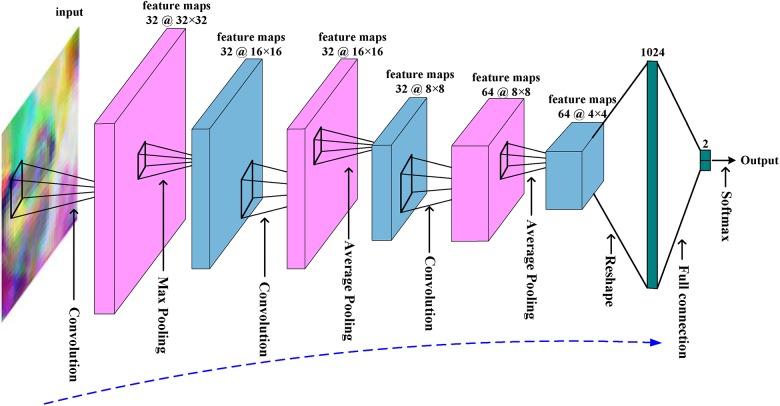
The overall architecture of the CNN used in this work.

The architecture of the CNN is summarized in Figure 4. The network has an input of 32 × 32 RGB patch. There are three convolutional layers and three pooling layers. The kernel size of convolutional layer is 5 × 5 with 2 pixels padding, and the kernel size and stride of pooling layers is 3 × 3 and 2. The input patch has a size of 32 × 32 and 3 RBG channels. The first convolutional layer generates 32 feature maps with a size of 32 × 32. After max pooling, these 32 feature maps were down-sampled into 16 × 16. The next two convolutional layers and average pooling layers finally generate 64 features maps with a size of 4 × 4. These features are concatenated as a feature vector, and then fed to full connection layer and softmax layer for classification. There are also rectified linear units layers and local response normalization layers in CNN, but are not shown for simplicity.

The CNN was trained with patches from NC and AD subjects, and there are 62967 (subject number 417 times 151) patches which are randomly split into 417 mini-batches. Mini-batch stochastic gradient descent was used to update the coefficients of CNN. In each step, a mini-batch was fed into CNN, and then error back propagation algorithm was carried out to computer gradient *g_j_* of *j*th coefficient θ*_j_*, and update the coefficient as θ*′_j_* = θ*_j_* + 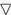
θ*n j*, in which 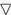
θ*n j* = *m*
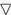
θ*n-1 j-* η(*g_j_* + λθ*_j_*) is the increment of θ*_j_* at *n*th step. The momentum *m*, learning rate η and weight decay λ are set as 0.9, 0.001, and 0.0001, respectively, in this work. It is called one epoch with all mini-batches used to train CNN once. The CNN was trained with 30 epochs. Once the network was trained, CNN will be used to extract high level features of MCI subjects’ images. The 1024 features output by the last pooling layer were taken as CNN-based features. Thus, CNN generates 154624 (1024 × 151) features for each image.

### FreeSurfer-Based Features

The FreeSurfer (version 4.3) (Fischl and Dale, 2000; Fischl et al., 2004; Desikan et al., 2006; Han et al., 2006) was used to mine more morphological information of MRI images, such as cortical volume, surface area, cortical thickness average, and standard deviation of thickness in each region of interest. These features can be downloaded directly from ADNI website, and 325 features are used to predict MCI-to-AD conversion after age correction. The age correction for FreeSurfer-based features is similar as described above, but on these 325 features instead of on intensity values of MRI images.

### Features Selection

Redundant features maybe exist among CNN-based features, thus we introduced the principle component analysis (PCA) (Avci and Turkoglu, 2009; Babaoğlu et al., 2010; Wu et al., 2013) and least absolute shrinkage and selection operator (LASSO) (Kukreja et al., 2006; Usai et al., 2009; Yamada et al., 2014) to reduce the final number of features.

PCA is an unsupervised learning method that uses an orthogonal transformation to convert a set of samples consisting of possibly correlated features into samples consisting of linearly uncorrelated new features. It has been extensively used in data analysis (Avci and Turkoglu, 2009; Babaoğlu et al., 2010; Wu et al., 2013). In this work, PCA is adopted to reduce the dimensions of features. Parameters of PCA are: (1) For CNN-based features, there are 1024 features for each patch. After PCA, *P_C_* features were left for each patch, since there are 151 patches for one subject, there are still *P_C_* × 151 features for each subject; (2) For FreeSurfer-based features, *P_F_* features were left for each MCI subject.

LASSO is a supervised learning method that uses *L_1_* norm in sparse regression (Kukreja et al., 2006; Usai et al., 2009; Yamada et al., 2014) as follows:

(1) $$\underset{\alpha }{\min}\;0.5∥y-D\alpha {∥}_{2}^{2}+\lambda ∥\alpha {∥}_{1}$$

Where ***y***∈**R**^1 × *N*^ is the vector consisting of *N* labels of training samples, **D**∈**R**^*N* × *M*^ is the feature matrix of *N* training samples consisting of *M* features, *λ* is the penalty coefficient that was set to 0.1, and **α**∈**R**^1 × *M*^ is the target sparse coefficients and can be used for selecting features with large coefficients. The LASSO was solved with least angle regression (Efron et al., 2004), and *L* features are selected after *L* iterations. Parameters of LASSO are: (1) For CNN-based features, *L_C_* features were selected from *P_C_* × 151 features for each MCI subject; (2) For FreeSurfer-based features, *L_F_* features were selected from *P_F_* features. After PCA and LASSO, there were *L_C_* + *L_F_* features.

**FIGURE 5:**
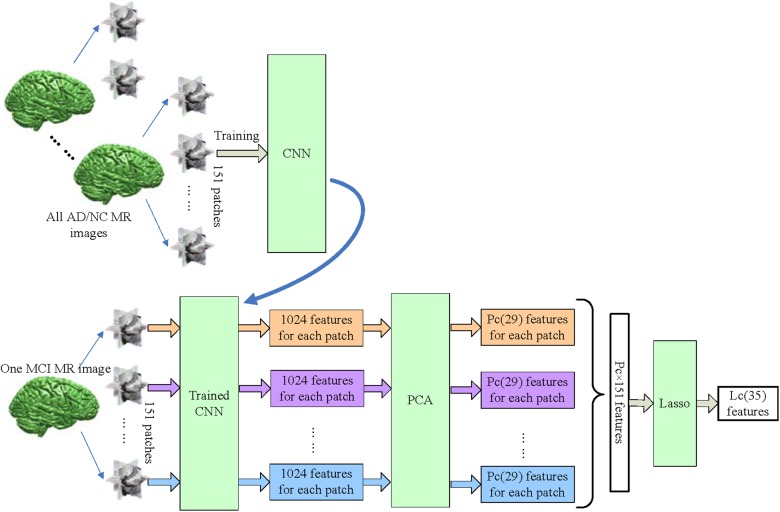
The workflow of extracting CNN-based features. The CNN was trained with all AD/NC patches, and used to extract deep features from all 151 patches of MCI subject. The feature number of each patch is reduced to *P_C_* (*P_C_* = 29) from 1024 by PCA. Finally, Lasso selects *L_C_* (*L_C_* = 35) features from *P_C_* × 151 features for each MCI subject.

Figure 5 shows more details of CNN-based features. 151 patches are extracted from all MRI images, including AD, NC, and MCI. First, the CNN is trained with patches of all AD and NC subjects. After that, the trained CNN is used to output 1024 features from each MCI patch. The 1024 features of each patch are reduced to *P_C_* features by PCA, and then features of all 151 patches from one subject are concatenated, and Lasso is used to select *L_C_* most informative features from them.

### Extreme Learning Machine

The extreme learning machine, a feed-forward neural network with a single layer of hidden nodes, learns much faster than common networks trained with back propagation algorithm (Huang et al., 2012; Zeng et al., 2017). A special extreme learning machine, that adopts kernel (Huang et al., 2012) to calculates the outputs as formula (2) and avoids the random generation of input weight matrix, is chosen to classify converters/non-converters with both CNN-based features and FreeSurfer-based features. In formula (2), the Ω is a matrix with elements Ω*_i_*_,_*_j_* = **K**(***x****_i_*, ***x****_j_*), where **K**(***a, b***) is a radial basis function kernel in this study, [***x***_1_,…, ***x***_***N***_] are N training samples, ***y*** is the label vector of training samples, and ***x*** is testing sample. *C* is a regularization coefficient and was set to 1 in this study.

(2) $$f(x)={\left[\begin{array}{c} K(x,\;x_{1})\\ \overset{\cdot }{\underset{\cdot }{\cdot }}\\ K(x,\;x_{N}) \end{array}\right]}^{T}{\left(\Omega +1/C\right)}^{T}y$$

### Implementation

In our implementation, CNN was accomplished with Caffe^1^, LASSO was carried out with SPAMS^2^, and extreme learning machine was performed with shared online code^3^. The hippocampus segmentation was implemented with MALPEM^4^ (Ledig et al., 2015) for all MRI images. Then all hippocampus masks were registered as corresponding MRI images, and then overlapped to create a mask containing hippocampus regions. All image features were normalized to have zero mean and unit variance before training or selection. To evaluate the performance, Leave-one-out cross validation was used as (Coupé et al., 2012; Ye et al., 2012; Zhang et al., 2012).

## Results

### Validation of the Robustness of 2.5D CNN

To validate the robustness of the CNN, several experiments have been performed with the CNN. In experiments, the binary decisions of CNN for 151 patches were united to make final diagnosis of the testing subject. We compared the performance in four different conditions: (1) The CNN was trained with AD/NC patches and used to classify AD/NC subjects; (2) The CNN was trained with converters/non-converters patches and used to classify converters/non-converters; (3) The CNN was trained with AD/NC patches and used to classify converters/non-converters; (4) The condition is similar with (3), but with different sampling patches in each validation run.

The results are shown in Table 2. The CNN has a poor accuracy of 68.49% in classifying converters/non-converters when trained with converters/non-converters patches, but CNN has obtained a much higher accuracy of 73.04% when trained with AD/NC patches. This means that the CNN learned more useful information from AD/NC data than that from converters/non-converters data. And the prediction performance of CNN is close when different sampling patches are used.

**Table 2:** The performance of the 2.5D CNN.

	Classifying: AD/NC Trained with: AD/NC	Classifying: MCIc/MCInc Trained with: MCIc/MCInc	Classifying: MCIc/MCInc Trained with: AD/NC	Different patch Sampling
Accuracy	88.79%	68.68%	73.04%	72.75%
Standard deviation	0.61%	1.63%	1.31%	1.20%
Confidence interval	[0.8862, 0.8897]	[0.6821, 0.6914]	[0.7265, 0.7343]	[0.7252, 0.7299]


*MCIc means MCI converters. MCInc means MCI non-converters. The results were obtained with 10-fold cross validations, and averaged over 50 runs.*

### Effect of Combining Two Types of Features

In this section, we present the performance of CNN-based features, FreeSurfer-based features, and their combinations. The *P_C_, P_F_, L_C_*, and *L_F_* parameters were set to 29, 150, 35, and 40, respectively, which were optimized in experiments. Finally, 75 features were selected and fed to the extreme learning machine.

**FIGURE 6:**
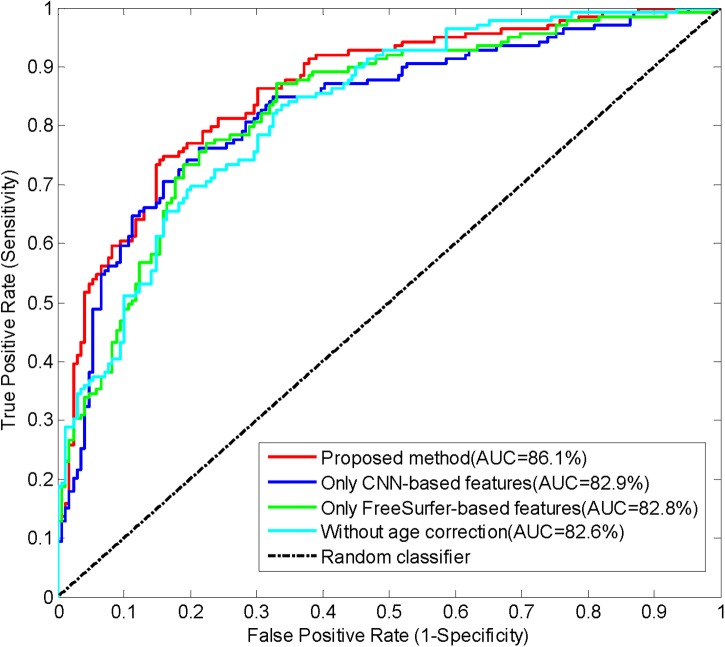
The ROC curves of classifying converters/non-converters when different features used or without age correction.

Performance was evaluated by calculating accuracy (the number of correctly classified subjects divided by the total number of subjects), sensitivity (the number of correctly classified MCI converters divided by the total number of MCI converters), specificity (the number of correctly classified MCI non-converters divided by the total number of MCI non-converters), and AUC (area under the receiver operating characteristic curve). The performances of the proposed method and the approach with only one type of features are summarized in Table 3. These results indicates that the approaches with only CNN-based features or FreeSurfer-based features have similar performances, and the proposed method combining both features achieved best accuracy, sensitivity, specificity and AUC. Thus, it is meaningful to combine two features in the prediction of MCI-to-AD conversion. The AUC of the proposed method reached 86.1%, indicating the promising performance of this method. The receiver operating characteristic (ROC) curves of these approaches are shown in Figure 6.

**Table 3:** The performance of different features used, and the performance without age correction.

Method	Accuracy	Sensitivity	Specificity	AUC
Proposed method (both features)	**79.9%**	**84%**	**74.8%**	**86.1%**
Only CNN-based features	76.9%	81.7%	71.2%	82.9%
Only FreeSurfer-based features	76.9%	82.2%	70.5%	82.8%
Without age correction	75.3%	79.9%	69.8%	82.6%


*Bold values indicate the best performance in each column.*

### Impact of Age Correction

We investigated the impact of age correction on the prediction of conversion here. The prediction accuracy in Table 3 and the ROC curves in Figure 6 implied that age correction can significantly improve the accuracy and AUC, Thus, age correction is an important step in the proposed method.

### Comparisons to Other Methods

In this section, we first compared the extreme learning machine with support vector machine and random forest. The performances of three classifiers are shown in Table 4, indicating that extreme learning machine achieves the best accuracy and AUC among three classifiers.

**Table 4:** Comparison of extreme learning machine with other two classifiers.

Method	Accuracy	Sensitivity	Specificity	AUC
SVM	**79.87%**	83.43%	**75.54%**	83.85%
Random forest	75.0%	82.84%	65.47%	81.99%
Extreme learning machine	**79.87%**	**84.02%**	74.82%	**86.14%**


*Implementation of SVM was performed using third party library LIBSVM (https://www.csie.ntu.edu.tw/~cjlin/libsvm/), and the random forest was utilized with the third party library (http://code.google.com/p/randomforest-matlab). Both classifiers used the default settings.*

Then we compared the proposed method with other state-of-the-art methods that use the same data (Moradi et al., 2015; Tong et al., 2017), which consists of 100 MCI non-converters and 164 MCI converters. In both methods, MRI images were first preprocessed and registered, but in different ways. After that, features selection was performed to select the most informative voxels among all MRI voxels. Moradi used regularized logistic regression algorithm to select a subset of MRI voxels, and Tong used elastic net algorithm instead. Both methods trained feature selection algorithms with AD/NC data to learn the most discriminative voxels and then used to selected voxels from MCI data. Finally, Moradi used low density separation to calculate MRI biomarkers and to predict MCI converters/non-converters. Tong used elastic net regression to calculate grading biomarkers from MCI features, and SVM was utilized to classify MCI converters/non-converters with grading biomarker.

For fair comparisons, both 10-fold cross validation and leave-one-out cross validation were performed on the proposed method and method of Tong et al. (2017) with only MRI data was used. Parameters of the compared approaches were optimized to achieve best performance. Table 5 shows the performances of three methods in 10-fold cross validation and Table 6 summarizes the performances in leave-one-out cross validations. These two tables demonstrate that the proposed method achieves the best accuracy and AUC among three methods, which means that the proposed method is more accurate in predicting MCI-to-AD conversion than other methods. The sensitivity of the proposed method is a little lower than the method of Moradi et al. (2015) but much higher than the method of Tong et al. (2017), and the specificity of the proposed method is between other two methods. Higher sensitivity means lower rate of missed diagnosis of converters, and higher specificity means lower rate of misdiagnosing non-converters as converters. Overall, the proposed method has a good balance between the sensitivity and specificity.

**Table 5:** Comparison with others methods on the same dataset in 10-fold cross validation.

Method	Accuracy	Sensitivity	Specificity	AUC
MRI biomarker in Moradi et al., 2015	74.7%	**88.9%**	51.6%	76.6%
Global grading biomarker in Tong et al., 2017	78.9%	76.0%	**82.9%**	81.3%
Proposed method	**79.5%**	86.1%	68.8%	**83.6%**


*The performances of MRI biomarker and global grading biomarker are described in Moradi et al. (2015) and (Tong et al., 2017). The results are averages over 100 runs, and the standard deviation/confidence intervals of accuracy and AUC of the proposed method are 1.19%/[0.7922, 0.7968] and 0.83%/[0.8358, 0.8391]. Bold values indicate the best performance in each column.*

**Table 6:** Comparison with others methods on the same dataset in leave-one-out cross validation.

Method	Accuracy	Sensitivity	Specificity	AUC
MRI biomarker in Moradi et al., 2015	–	–	–	–
Global grading biomarker in Tong et al., 2017	78.8%	76.2%	**83%**	81.2%
Proposed method	**81.4%**	**89.6%**	68%	**87.8%**


*The global grading biomarkers was download from the web described in Tong et al. (2017) and the experiment was performed with same method as in Tong et al. (2017). Bold values indicate the best performance in each column.*

## Discussion

The CNN has a better performance when trained with AD/NC patches rather than MCI patches, we think the reason is that the pathological changes between MCI converters and non-converters are slighter than those between AD and CN. Thus, it is more difficult for CNN to learn useful information directly from MCI data about AD-related pathological changes than from AD/NC data. The pathological changes are also hampered by inter-subject variations for MCI data. Inspired by the work in Moradi et al. (2015) and Tong et al. (2017) which use information of AD and NC to help classifying MCI, we trained the CNN with the patches from AD and NC subjects and improved the performance.

After non-rigid registration, the differences between all subject’s MRI brain image are mainly in hippocampus (Tong et al., 2017). So we extracted 2.5D patches only from hippocampus regions, that makes the information of other regions lost. For this reason, we included the whole brain features calculated by FreeSurfer as complementary information. The accuracy and AUC of classification are increased to 79.9 and 86.1% from 76.9 to 82.9% with the help of FreeSurfer-based features. To explore which FreeSurfer-based features contribute mostly when they are used to predict MCI-to-AD conversion, we used Lasso to select the most informative features, and the top 15 features are listed in Table 7, in which the features are almost volume and thickness average of regions related to AD. The thickness average of frontal pole is the most discriminative feature. The quantitative features of hippopotamus are not listed, indicating they contribute less than these listed features when predicting conversion. The CNN extract the deep features of hippopotamus morphology, rather than the quantitative features of hippopotamus, which are discriminative for AD diagnosis. Therefore, The CNN-based features and FreeSurfer-based features contain different useful information for classification of converters/non-converters, and they are complementary to improve the performance of classifier.

**Table 7:** The 15 most informative FreeSurfer-based features for predicting MCI-to-AD conversion.

Number	FreeSurfer-based feature
1	Cortical Thickness Average of Left FrontalPole
2	Volume (Cortical Parcellation) of Left Precentral
3	Volume (Cortical Parcellation) of Right Postcentral
4	Volume (WM Parcellation) of Left AccumbensArea
5	Cortical Thickness Average of Right CaudalMiddleFrontal
6	Cortical Thickness Average of Right FrontalPole
7	Volume (Cortical Parcellation) of Left Bankssts
8	Volume (Cortical Parcellation) of Left PosteriorCingulate
9	Volume (Cortical Parcellation) of Left Insula
10	Cortical Thickness Average of Left SuperiorTemporal
11	Cortical Thickness Standard Deviation of Left PosteriorCingulate
12	Volume (Cortical Parcellation) of Left Precuneus
13	Volume (WM Parcellation) of CorpusCallosumMidPosterior
14	Volume (Cortical Parcellation) of Left Lingual
15	Cortical Thickness Standard Deviation of Right Postcentral


Different from the two methods used in Moradi et al. (2015) and Tong et al. (2017), which directly used voxels as features, the proposed method employs CNN to learn the deep features from the morphology of hippopotamus, and combined CNN-based features with the globe morphology features that were computed by FreeSurfer. We believe that the learnt CNN features might be more meaningful and more discriminative than voxels. When comparing with these two methods, only MRI data was used, but the performances of these two methods were improved when combined MRI data with age and cognitive measures, so investigating the combination of the propose approach with other modality data for performance improvement is also one of our future works.

We have also listed several deep learning-based studies in recent years for comparison in Table 8. Most of them have an accuracy of predicting conversion above 70%, especially the last three approaches (including the proposed one) have the accuracy above 80%. The best accuracy was achieved by Lu et al. (2018a), which uses both MRI and PET data. However, when only MRI data is used, Lu’s method declined the accuracy to 75.44%. Although an accuracy of 82.51% was also obtained with PET data (Lu et al., 2018b), PET scanning usually suffers from contrast agents and more expensive cost than the routine MRI. In summary, our approach achieved the best performance when only MRI images were used and is expected to be improved by incorporating other modality data, e.g., PET, in the future.

**Table 8:** Results of previous deep learning based approaches for predicting MCI-to-AD conversion.

Study	Number of MCIc/MCInc	Data	Conversion time	Accuracy	AUC
Li et al., 2015	99/56	MRI + PET	18 months	57.4%	–
Singh et al., 2017	158/178	PET	–	72.47%	–
Ortiz et al., 2016	39/64	MRI + PET	24 months	78%	82%
Suk et al., 2014	76/128	MRI + PET	–	75.92%	74.66%
Shi et al., 2018	99/56	MRI + PET	18 months	78.88%	80.1%
Lu et al., 2018a	217/409	MRI + PET	36 months	**82.93%**	–
Lu et al., 2018a	217/409	MRI	36 months	75.44%	–
Lu et al., 2018b	112/409	PET	–	82.51%	–
This study	164/100	MRI	36 months	81.4%	**87.8%**


*MCIc means MCI converters. MCInc means MCI non-converters. Different subjects and modalities of data are used in these approaches. All the criteria are copied from the original literatures. Bold values indicate the best performance in each column.*

In this work, the period of predicting conversion was set to 3 years, that separates MCI subjects into MCI non-converters and MCI converters groups by the criterion who covert to AD within 3 years. But not matter what the period for prediction is, there is a disadvantage that even the classifier precisely predict a MCI non-converters who would not convert to AD within a specific period, but the conversion might still happen half year or even 1 month later. Modeling the progression of AD and predicting the time of conversion with longitudinal data are more meaningful (Guerrero et al., 2016; Xie et al., 2016). Our future work would investigate the usage of CNN in modeling the progression of AD.

## Conclusion

In this study, we have developed a framework that only use MRI data to predict the MCI-to-AD conversion, by applying CNN and other machine learning algorithms. Results show that CNN can extract discriminative features of hippocampus for prediction by learning the morphology changes of hippocampus between AD and NC. And FreeSurfer provides extra structural brain image features to improve the prediction performance as complementary information. Compared with other state-of-the-art methods, the proposed one outperforms others in higher accuracy and AUC, while keeping a good balance between the sensitivity and specificity.

## Author Contributions

WL and XQ conceived the study, designed the experiments, analyzed the data, and wrote the whole manuscript. TT and QG provided the preprocessed data. WL, XQ, DG, XD, and MX carried out experiments. YY and GG helped to analyze the data and experiments result. MD and XQ revised the manuscript.

## Conflict of Interest Statement

The authors declare that the research was conducted in the absence of any commercial or financial relationships that could be construed as a potential conflict of interest.

## Footnotes

1 http://caffe.berkeleyvision.org/

2 http://spams-devel.gforge.inria.fr/

3 http://www.ntu.edu.sg/home/egbhuang/

4 http://www.christianledig.com/
